# Assessment of glenoid bone loss and other osseous shoulder pathologies comparing MR-based CT-like images with conventional CT

**DOI:** 10.1007/s00330-023-09939-9

**Published:** 2023-07-15

**Authors:** Georg C. Feuerriegel, Sophia Kronthaler, Kilian Weiss, Bernhard Haller, Yannik Leonhardt, Jan Neumann, Daniela Pfeiffer, Nina Hesse, Bernd Erber, Benedikt J. Schwaiger, Marcus R. Makowski, Klaus Woertler, Dimitrios C. Karampinos, Markus Wurm, Alexandra S. Gersing

**Affiliations:** 1 Department of Radiology, Klinikum Rechts der Isar, School of Medicine, Technical University of Munich, Ismaninger Strasse 22, 81675 Munich, Germany; 2 Philips GmbH Market DACH, Hamburg, Germany; 3 Institute of Medical Informatics, Statistics and Epidemiology, School of Medicine, Technical University of Munich, Munich, Germany; 4 Musculoskeletal Radiology Section, Klinikum Rechts der Isar, School of Medicine, Technical University of Munich, Munich, Germany; 5 Department of Radiology, University Hospital of Munich, LMU Munich, Munich, Germany; 6 Department of Neuroradiology, Klinikum Rechts der Isar, School of Medicine, Technical University of Munich, Munich, Germany; 7 Department of Trauma Surgery, Klinikum Rechts der Isar, School of Medicine, Technical University of Munich, Munich, Germany; 8 Department of Neuroradiology, University Hospital of Munich, LMU Munich, Munich, Germany

**Keywords:** Bankart lesion, Shoulder dislocation, Fracture, Bone

## Abstract

**Objectives:**

To evaluate and compare the diagnostic performance of CT-like images based on a 3D T1-weighted spoiled gradient-echo sequence (T1 GRE), an ultra-short echo time sequence (UTE), and a 3D T1-weighted spoiled multi-echo gradient-echo sequence (FRACTURE) with conventional CT in patients with suspected osseous shoulder pathologies.

**Materials and methods:**

Patients with suspected traumatic dislocation of the shoulder (*n *= 46, mean age 40 ± 14.5 years, 19 women) were prospectively recruited and received 3-T MR imaging including 3D T1 GRE, UTE, and 3D FRACTURE sequences. CT was performed in patients with acute fractures and served as standard of reference (*n *= 25). Agreement of morphological features between the modalities was analyzed including the glenoid bone loss, Hill-Sachs interval, glenoid track, and the anterior straight-line length. Agreement between the modalities was assessed using Bland-Altman plots, Student’s *t*-test, and Pearson’s correlation coefficient. Inter- and intrareader assessment was evaluated with weighted Cohen’s *κ* and intraclass correlation coefficient.

**Results:**

All osseous pathologies were detected accurately on all three CT-like sequences (*n *= 25, *κ *= 1.00). No significant difference in the percentage of glenoid bone loss was found between CT (mean ± standard deviation, 20.3% ± 8.0) and CT-like MR images (FRACTURE 20.6% ± 7.9, T1 GRE 20.4% ± 7.6, UTE 20.3% ± 7.7, *p *> 0.05). When comparing the different measurements on CT-like images, measurements performed using the UTE images correlated best with CT.

**Conclusion:**

Assessment of bony Bankart lesions and other osseous pathologies was feasible and accurate using CT-like images based on 3-T MRI compared with conventional CT. Compared to the T1 GRE and FRACTURE sequence, the UTE measurements correlated best with CT.

**Clinical relevance statement:**

In an acute trauma setting, CT-like images based on a T1 GRE, UTE, or FRACTURE sequence might be a useful alternative to conventional CT scan sparing associated costs as well as radiation exposure.

**Key Points:**

*• No significant differences were found for the assessment of the glenoid bone loss when comparing measurements of CT-like MR images with measurements of conventional CT images.*

*• Compared to the T1 GRE and FRACTURE sequence, the UTE measurements correlated best with CT whereas the FRACTURE sequence appeared to be the most robust regarding motion artifacts.*

*• The T1 GRE sequence had the highest resolution with high bone contrast and detailed delineation of even small fractures but was more susceptible to motion artifacts.*

**Supplementary Information:**

The online version contains supplementary material available at 10.1007/s00330-023-09939-9.

## Introduction

Dislocation of the shoulder joint is the most common major joint dislocation with approximately 95% being anterior shoulder dislocations [1, 2]. In many cases, the dislocation causes the avulsion of anterior bony and/or soft tissue structures (Bankart lesion) due to the forceful external rotation of the abducted arm which levers the humeral head out of its socket [2]. In clinical routine, the glenoid bone loss (GBL) and type of bone loss (on-track/off-track) are important factors for the decision regarding the treatment strategy, since the type of surgical procedure relies on the extent of the GBL and type of Hill-Sachs Lesion (HSL) [3]. Procedures such as remplissage and inferior capsular shift are recommended for a GBL between 13.5 and 25%, whereas a bone block transfer is usual recommended for GBL greater than 20–25% [4]. Therefore, accurate assessment of the osseous structure is essential for therapy planning. In an acute trauma setting, conventional CT is part of the clinical routine work up to assess osseous pathologies for potential surgical planning with the disadvantage of using ionizing radiation. Recent studies proposed the application of 3D MRI for the assessment of osseous defects of the glenoid and humeral head, which showed to be comparable with 3D CT scans [5, 6]. The reliable assessment of osseous and soft tissue structures using one modality would be beneficial, since this approach would save costs and time for the final diagnosis and therapy planning and reduce the exposure to radiation. In contrast to soft tissues, which have long transverse relaxation times, MR imaging of osseous structures is challenging due to the low proton density and short or ultrashort T2/T2* decay times [7]. Several approaches have been proposed in previous studies in order to create reliable CT-like MR images with bright bone-like contrast [8–16].

3D gradient echo sequences such as the 3D T1 GRE and the FRACTURE sequence have been successfully used to detect and assess vertebral fractures as well as fractures located at the extremities and were able to assess the destruction pattern of benign and malignant bone tumors [10, 17–19].

UTE and zero echo time (ZTE) sequences were successfully applied for quantification of glenoid bone loss and vertebral fractures in comparison to 2D and 3D conventional CT [17, 19]. Due to the ability of UTE and ZTE to detect the fast-decaying signal of solid tissues, the techniques have an excellent bone to soft tissue contrast by relying on the inversion of the proton density contrast between bone and surrounding soft tissues [20, 21]. The purpose of this study was to assess and compare the diagnostic performance of conventional CT and of the three most promising CT-like MR-based images using 3D T1 GRE, UTE, and 3D FRACTURE sequences in patients with traumatic shoulder dislocation and suspected osseous lesions.

## Methods and materials

### Patient selection

Patients admitted to the emergency department with suspected traumatic dislocation of the shoulder between January 2021 and July 2022 were included in the study (Table 1). All participants underwent 3-T MRI of the shoulder within 2 days after trauma, and in patients with fractures a CT examination was commenced as part of the diagnostic workup in clinical routine. The study was approved by our institutional review board. Written informed consent was obtained from all study participants prior to inclusion.

**Table 1:** Patient characteristics and pathologies assessed with conventional CT

Clinical characteristics	Total (%)
Number of patients	46 (100)
Age in years, mean ± SD	40 ± 14.5
Number of women	19 (41)
Number of men	27 (50)
Right side affected	28 (61)
Left side affected	18 (39)
Pathologies detected	
Acute anterior shoulder dislocation	38 (83)
Bankart lesion	30 (65)
Bony Bankart lesions	20 (43)
Greater tubercle fracture	4 (9)
Scapula fracture	1 (2)

### MR imaging

Each participant received MR imaging using a clinical routine shoulder protocol. Additionally, sagittal UTE, 3D T1 GRE, and 3D FRACTURE sequences were acquired for CT-like bone contrast.

The UTE sequence was based on a slab selective excitation and radial stack of stars readout starting in the center of k-space. A partial Fourier factor of 0.8 was used in phase encoding direction to reduce acquisition time. The 3D T1 GRE sequence was based on slab selective excitation in combination with Cartesian sampling and partial Fourier encoding in read out direction to keep the echo time short. Finally the 3D FRACTURE sequence [10] was based on a Cartesian multi-echo 3D T1 GRE sequence with four echoes acquired. During postprocessing, a magnitude summation of all echoes was performed and afterwards the last echo was subtracted to create a CT-like bone contrast. Both the 3D T1 GRE and the 3D FRACTURE sequences were accelerated using Compressed SENSE. Detailed scan parameters of the CT-like MR-based sequences are displayed in Table 2. To achieve CT-like bone contrast of the three CT-like MRI sequences, all image intensities were inverted after image reconstruction.

**Table 2:** Scan parameters of the sequences used in this study

Sequence	T1 GRE	FRACTURE	UTE
Echo time (ms)	1.9	2.3	0.19
Echo time increment (ms)	-	2.3	-
Repetition time (ms)	10	15.4	7.5
Flip angle (°)	8	15	5
Acceleration factor	4	4	1
Field of view (mm^3^)	150 × 150 × 80	150 × 150 × 80	150 × 150 × 100
Voxel size (acquisition, mm^3^)	0.4 × 0.4 × 0.4	0.45 × 0.45 × 0.45	0.45 × 0.45 × 1.5
Voxel size (reconstructed, mm^3^)	0.22 × 0.22 × 0.2	0.3 × 0.3 × 0.22	0.34 × 0.34 × 1
Acquisition time (min)	4:37	4:41	4:53

All participants were examined using a 3-T MR scanner (Ingenia Elition X; Philips Healthcare) with a dedicated 16-channel shoulder coil (dStream shoulder 16ch coil, Philips Healthcare). UTE images were acquired in the sagittal plane. Due to the isotropic acquisition voxel size, the T1 GRE and FRACTURE sequences were acquired in axial orientation und reformatted in the sagittal and coronal plane as well as inverted to resemble a bright CT-like bone contrast.

### CT examinations

Participants with traumatic fracture of the shoulder underwent CT using either an IQon Spectral CT scanner (Philips Healthcare) or a Siemens Somatom go.Top scanner (Siemens Healthineers). Clinical scan parameters were set according to the clinical routine: collimation, 0.6 mm; pixel spacing, 0.56 mm; pitch factor, 0.8; tube voltage (peak), 120 kV; and modulated tube current, 102–132 mA. Images were acquired in axial orientation and reformatted in sagittal and coronal orientation using a bone-specific convolution kernel (170H/YB, 3-mm slices).

### Image analysis

MR and CT images were read by two radiologists (an attending musculoskeletal radiologist with 10 years of experience and a resident with 4 years of experience in musculoskeletal radiology). Image analysis was performed on a picture archiving and communication system (PACS) work station certified for clinical use (IDS7 21.2, Sectra). The different CT-like MR images and conventional CT images were analyzed separately and independently in random order and blinded to clinical information and all other imaging data. The CT-like MR images and conventional CT images were read with at least 8 weeks in between readings, respectively. The different CT-like MR images (3D T1 GRE, FRACTURE, and UTE) were assessed separately with at least 2 weeks in between the readings, respectively. For intrareader reproducibility, the conventional CT and CT-like MR images of 10 participants were assessed once again after 8 weeks by both radiologists.

### Quantitative and semi-quantitative parameters

**Fig. 1:**
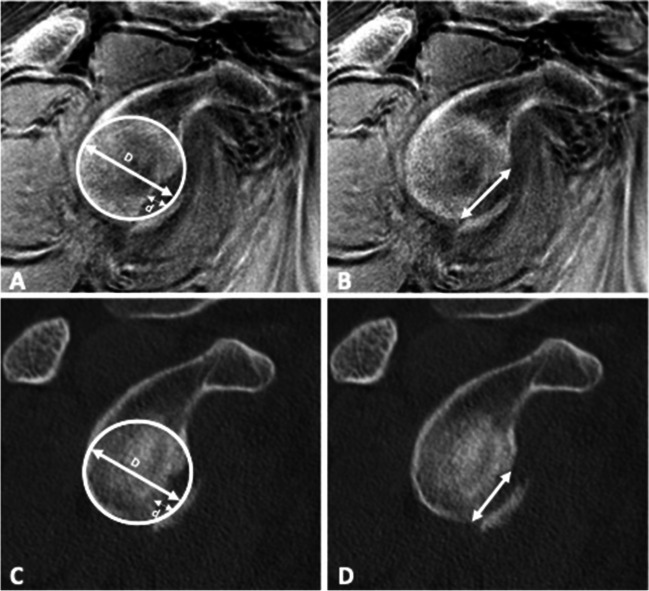
Evaluation of the glenoid bone loss using the best fit method on a sagittal CT-like UTE image (**A**) and conventional CT (**C**). A circle was drawn by one reader along the posterior, anterior, and inferior margin of the glenoid surface, and the percentage of bone loss was calculated by dividing the width of the anterior bone loss with the width of the circle. The glenoid track is calculated as 0.83 *D* − *d*, in which *D* represents the diameter of the intact glenoid in millimeters and the *d* corresponds to the amount of glenoid bone loss in millimeters [24]. In this case, the glenoid bone loss was 8.75 % (**B** and** D**). The anterior straight line measuring the loss of convexity of the anterior glenoid surface after shoulder dislocation with a bony Bankart lesion. A very strong correlation with CT measurements was found in this case (*r* = 0.97)

**Fig. 2:**
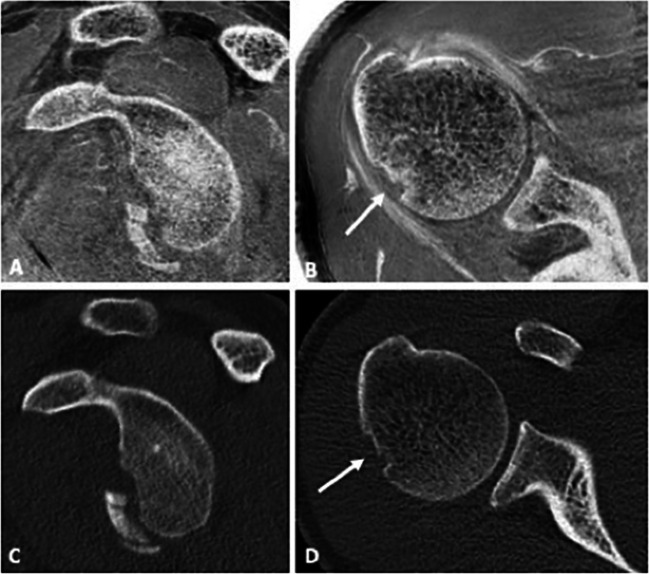
A 22-year-old male patient with acute anterior shoulder dislocation. **A** Sagittal intensity-inverted FRACTURE image showing the glenoid defect of the anterior margin with adjacent fracture fragments. The glenoid track in this case is 7.81 mm. **B** Axial FRACTURE MR image showing a small superficial Hill-Sachs lesion. The Hill-Sachs interval measured 18.1 mm. The Hill-Sachs interval is less than the glenoid track; thus, findings are characterized as on-track lesion with a low risk of engagement. **C** Sagittal and **D** axial conventional CT of the same patient showing no significant differences of the measurements of the glenoid track and Hill-Sachs index when compared to the MR-based measurements. In this case, a strong correlation between the measurements on CT and CT-like MRI was found (*r* = 0.93)

**Fig. 3:**
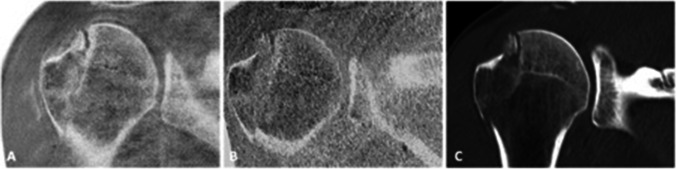
Fracture dislocation with avulsion of the greater tubercle in a 23-year-old patient admitted to the ER. The fracture was equally detectable using a FRACTURE sequence (**A**) as well as a T1 GRE sequence (**B**) compared to the conventional CT (**C**)

Images were analyzed for the presence of bony Bankart lesions and Hill-Sachs defects. Furthermore, the images were analyzed for further osseous pathologies including scapula fractures, subchondral cysts, calcific tendonitis, and degenerative changes of the AC-joint. The percentage of glenoid bone loss was evaluated in participants with bony Bankart lesions using the best fit model on sagittal images [22]. A circle was drawn using the anterior, posterior, and inferior margin of the glenoid surface as outer boundary and the percentage of bone loss was calculated by dividing the width of the anterior bone loss with the diameter of the circle (Fig. 1). Additionally, anterior glenoid loss leads to a loss of the convexity of the anterior glenoid which was evaluated by drawing and measuring an anterior straight line [23]. The humerus was assessed for Hill-Sachs lesions and in patients with a bipolar lesion, the glenoid track was calculated as previously reported (Fig. 2) [24]. Fractures of the humeral head were graded according to the international AO and Neer classification (Fig. 3) [25, 26]. Furthermore, the glenoid bone width was measured in the sagittal plane.

Image quality of the CT-like MR images and overall diagnostic confidence were evaluated using a 5-point Likert scale mainly evaluating the overall diagnostic accuracy and appearance of an image: 1 (poor) definite diagnosis is impossible due to major inhomogeneity, 2 (below average) evaluation of major findings is possible but still major inhomogeneities, 3 (fair) adequate diagnosis possible, moderate inhomogeneities, 4 (good) definite diagnosis possible, minimal inhomogeneities, 5 (excellent) exact diagnosis possible with no inhomogeneities. Motion artifacts including blurring, streaking, and shading due to voluntary or involuntary movement were graded using a 4-point scale (1= severe artifacts, adequate diagnosis impossible; 2= moderate artifacts, evaluation of major findings possible; 3= little artifacts, definite diagnosis possible; 4= no artifacts, exact diagnosis possible).

### Statistics

Statistics were performed by an experienced biostatistician. Bland-Altman plots were created to compare the agreement between measurements on CT and each CT-like MR sequence, respectively. Additionally, Student’s *t*-test was used to test for differences in the mean glenoid bone width, the anterior straight-line length, and length of the Hill-Sachs interval in order to assess presence of systematic differences of the mean. Correlation of the percentage of glenoid bone loss between MRI and CT was performed using Pearson’s correlation. The Wilcoxon signed-rank test was used to calculate differences in diagnostic image quality between the three MR-derived CT-like assessed using the Likert scale. The inter- and intraobserver reliability was calculated using interclass correlation coefficient (ICC) and weighted Cohen’s *κ* [27, 28]. All statistical tests were performed two-sided and a level of significance (*α*) of 0.05 was used. The data were analyzed using IBM SPSS Statistics for Windows, version 27.0 (IBM Corporation).

## Results

In total, 46 patients (mean age 40 ± 14.5 years, 19 women) were included into the study (Table 1). Of the 46 participants with suspected traumatic shoulder dislocation, 30 patients were diagnosed with a traumatic Bankart lesion and 20 of these patients showed a bony Bankart lesion of the glenoid, using CT as the standard of reference.

### Glenoid bone loss

**Fig. 4:**
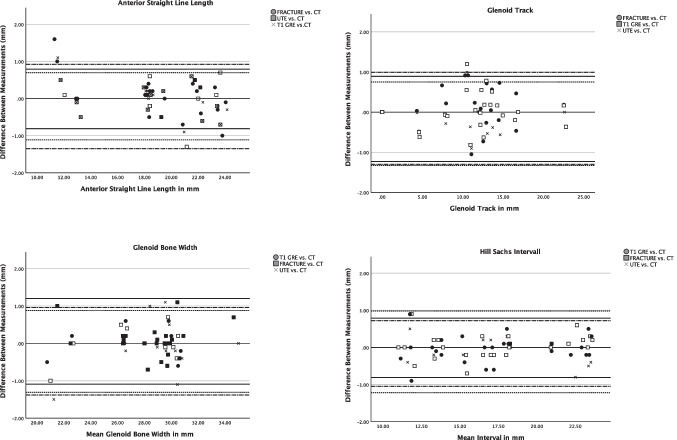
Bland-Altman plots for the agreement between measurements on the CT-like MRI sequences T1 GRE/UTE/FRACTURE and CT images, respectively. Upper and lower limits of agreement for the measurements on T1GRE vs. CT images are marked with fine dashed lines, respectively. Upper and lower limits of agreement for UTE vs. CT are marked with straight lines, respectively. The upper and lower limits of agreement for FRACTURE vs. CT are marked with alternating dashed and dotted lines, respectively

**Fig. 5:**
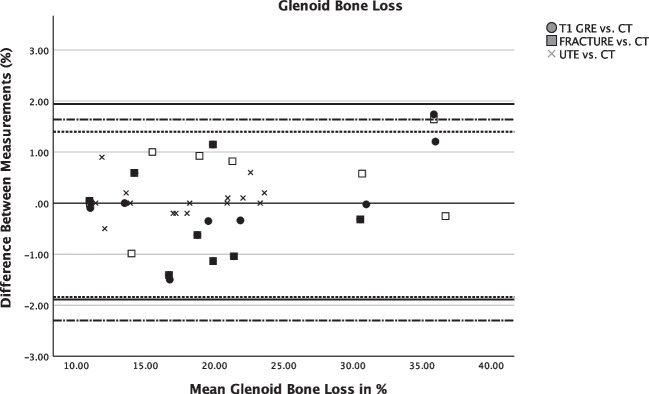
Agreement between measurements of the glenoid bone loss on the CT-like MRI sequences T1 GRE/UTE/FRACTURE and CT images depicted by a Bland-Altman plot. Upper and lower limits of agreement for the measurements on T1 GRE vs. CT images are marked with fine dashed lines. Upper and lower limits of agreement for UTE vs. CT are marked with straight lines. The upper and lower limits of agreement for FRACTURE vs. CT are marked with alternating dashed and dotted lines

All bony Bankart lesions (*N *= 20, 43%) were accurately detected by both readers on all CT-like MR images (readers 1 and 2: *κ* 1.00) (95% confidence interval 1.00–1.00). Good agreement between the measurements on CT and CT-like MRI was seen in the Bland-Altman plots (Figs. 4 and 5). Measurements of the percentage of glenoid loss on the CT-like MRI did not differ significantly compared to the gold standard CT (mean differences, FRACTURE 0.14%, T1 GRE 0.37%, and UTE 0.02% glenoid loss). The 95% limits of agreement showed that the range of discrepancies for the percentage of glenoid loss was comparable between the CT-like MR sequences (95% limits of agreement: UTE −1.9 to 1.9%, T1 GRE −1.8 to 1.4%, and FRACTURE −2.1 to 1.6%).

There was no significant difference in the percentage of glenoid bone loss between CT (mean ± standard deviation, 20.3 % ± 8.0, range 9–36%) and CT-like MR images (FRACTURE 20.6% ± 7.9, *p *= 0.85; T1 GRE 20.4% ± 7.6, *p *= 0.80; UTE 20.3% ± 7.7, range 8–36%, *p *= 0.86; Table 3). The glenoid track was correctly assessed on all CT-like MR images by both readers (off-track *n *= 6, on-Track *n *= 14, *κ* 1.00). No significant differences were found for the length of the Hill-Sachs interval between CT (mean ± standard deviation, 17.4 mm ± 4.1) and CT-like MR images (FRACTURE 17.3 mm ± 4.1, *p *= 0.89; T1 GRE 17.4 mm ± 4.2, *p *= 0.91; UTE 17.4 mm ± 4.2, *p *= 0.98).

**Table 3:** Mean measurements of the CT-like T1 GRE, FRACTURE, and UTE sequences compared to conventional CT

	CT	T1 GRE	FRACTURE	UTE	*p*-value
Glenoid bone loss (% ± SD)	20.3 ± 8.0	20.4 ± 7.6	20.6 ± 7.9	20.3 ± 7.7	0.962^*^
Anterior straight line (mm ± SD)	19.2 ± 3.6	19.1 ± 3.8	19.1 ± 3.9	19.2 ± 3.7	0.979^*^
Hill-Sachs interval (mm ± SD)	17.4 ± 4.1	17.4 ± 4.2	17.3 ± 4.1	17.4 ± 4.2	0.988^*^
Glenoid track (mm ± SD)	8.2 ± 7.1	8.1 ± 7.1	8.1 ± 7.1	8.2 ± 7.1	0.985^*^
Glenoid bone width (mm ± SD)	22.1 ± 3.8	21.5 ± 5.9	21.0 ± 5.9	22.1 ± 3.7	0.560^*^

*SD*, standard deviation; ^*^Student’s *t*-test using *p* < 0.05

Compared to conventional CT, no significant differences were found for the measurements of the anterior straight-line length (CT mean 19.2 mm ± 3.6) on all CT-like MR images (T1 GRE mean ± standard deviation 19.1 mm ± 3.8, *p *= 0.84, FRACTURE 19.1 mm ± 3.9, *p *= 0.78, UTE 19.2 mm ± 3.7, *p *= 0.94). When comparing the mean measurements of all CT-like MR sequences with conventional CT, there was no significant difference detected (Table 3).

### Diagnostic performance of CT-like MR sequences

All CT-like MR sequences showed a strong correlation with CT regarding the percentage of glenoid bone loss showing no substantial differences between each other (T1 GRE (*r* = 0.94, *p* < 0.001), FRACTURE (*r* = 0.91, *p* < 0.001), and UTE (*r* = 0.98, *p* < 0.001)). No significant differences were found between the CT-like sequences for the mean measurements of the glenoid width, glenoid loss, anterior straight-line length, and length of the Hill-Sachs interval (*p* > 0.05). The FRACTURE sequence was overall evaluated with the least motion artifacts measured on a 4-point scale (mean ± standard deviation, 3.6 ± 0.7, *p *= 0.46) compared to the UTE (3.2 ± 0.7, *p *= 0.64) and the T1 GRE sequence (2.9 ± 0.8, *p *= 0.73). The mean image quality measured on a 5-point Likert scale for all CT-like MR sequences was rated excellent with no significant differences between the sequences (FRACTURE 4.2 ± 0.7, *p *= 0.83, T1 GRE 4.1 ± 0.7, *p *= 0.24, UTE 4.2 ± 0.7, *p *= 0.69). The overall mean of the diagnostic confidence for all CT-like MR images was excellent (mean ± standard deviation, 4.2 ± 0.8).

### Inter- and intrareader agreement

A substantial to almost perfect interreader agreement was found for the measurements of the glenoid loss on CT-like MRI and conventional CT (ICC (range) 0.91–0.98 [95% confidence interval (CI) 0.87–0.99]). After 8 weeks, the images of 10 patients were assessed once more and all osseous pathologies were correctly identified again by both readers (readers 1 and 2: *κ* 1.00) (95% CI (1.00–1.00), both). The intrareader agreement for the measurements of the glenoid loss was substantial to almost perfect (ICC 0.98 [95% CI 0.94–0.99]).

## Discussion

The diagnosis and evaluation of bony Bankart lesions and other osseous pathologies of the shoulder were reliable and accurate on CT-like MR-based images acquired using 3D T1 GRE, UTE, and FRACTURE sequences compared to conventional CT. The gradient echo sequences T1 GRE and FRACTURE produced high-resolution 3D datasets with the FRACTURE sequence being more robust to motion artifacts compared to the 3D T1 GRE and UTE. Using the UTE sequence, the depiction of the cortical bone was correlated the highest with the conventional CT regarding the measurements of glenoid bone loss.

On conventional MR imaging, precise delineation of the bone margins is difficult due to the relatively long echo times and the limited ability to encode the short T2/T2* decay of bone [7]. The CT-like MR sequences used in this study are among the most established sequences for imaging of osseous tissue and were specifically created to have high bone to soft tissue contrast and to have a high resolution within cortical bone in order to detect even small fractures. Gradient echo sequences like, e.g., in “black bone” imaging have been developed to produce a high bone to soft tissue contrast by reducing the soft tissue contrast itself using an optimized low flip angle and a set echo time and repetition time [30]. Furthermore, UTE and ZTE sequences have the ability to detect the fast-decaying signal of solid tissues due to the short echoes and therefore can directly depict cortical bone [7, 31]. No significant differences in the assessment of glenoid bone loss were detected between conventional CT and the three most promising CT-like MR sequences. Additionally, the Bland-Altman analysis showed that the variability of the differences between CT and CT-like MRI was in a clinically acceptable range indicating that CT-like MRI may enable a reliable preoperative planning without the use of radiation dependent techniques. As most of the patients receive a preoperative MRI in order to assess injuries to soft tissue structures, simultaneous assessment of osseous structures would be beneficial to save time as well as costs and reduce radiation exposure to the patient.

The 3D T1 GRE sequence produced high-resolution images of the shoulder and was able to show clear fracture borders and small bone fragments even in cases with joint effusion. Most likely due to its high spatial resolution, the T1 GRE sequence was more sensitive to motion artifacts compared to the FRACTURE sequence which relies on the acquisition and summation of several echo images and therefore is less sensitive to motion artifacts. Furthermore, the high isotropic resolution of the T1GRE sequence makes the sequence more susceptible to Rician noise. Reducing the resolution of the T1 GRE sequence to a certain degree which reduces the SNR or applying DL-based denoising methods might increase the robustness regarding motion artifacts and image noise and should be analyzed in further investigations.

UTE and FRACTURE sequences were compared in a recent postmortem study regarding the detection of skull fractures, and both sequences were able to accurately detect skull fractures with a high accuracy. In contrast to our study, the resolution of the images in the previous study was lower, which may impair the detection of small fractures and fragments and moreover, image acquisition was performed postmortem [19]. In our study, we were able to perform reliable measurements of the glenoid in vivo using high resolution, enabling the detection of even small osseous lesions. In a recent case study, the FRACTURE sequence was used for fracture detection of various bones and joints. Yet, in contrast to our study, no quantitative or semi-quantitative measurements were acquired [10]. In our investigation, the FRACTURE sequence appeared to be the most robust regarding motion artifacts, most likely due to the acquisition of multiple gradient echoes and subsequent image summation. In an acute trauma setting, this might be a good alternative to conventional CT, especially if patients are not able to lie motionless due to pain or claustrophobia. Compared to the T1 GRE sequence, image resolution was slightly lower in the FRACTURE sequence and due to the stronger T2* weighting, especially of later echoes, not only bone but also menisci and ligaments appeared brighter on the inverted images, making the differentiation of bone and ligament structures difficult which may impair the fracture detection, e.g., in juxta-articular and avulsion fractures.

**Fig. 6:**
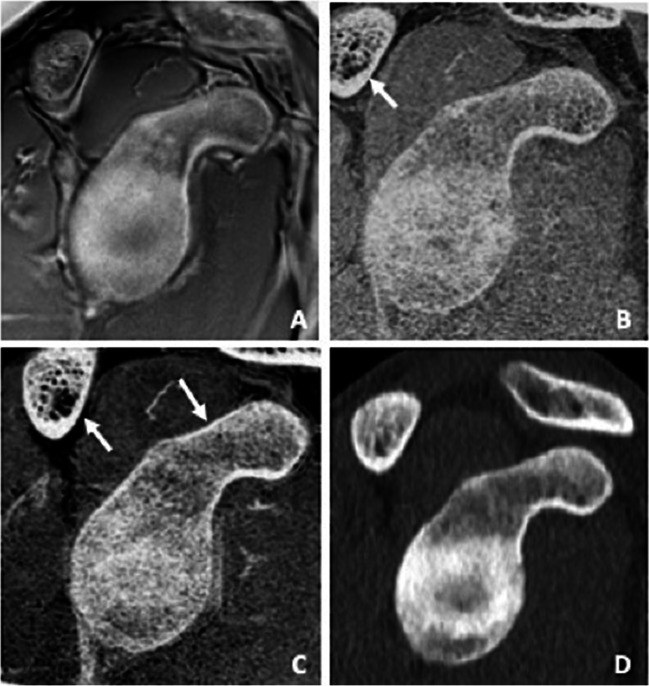
Intact glenoid of an 18-year-old patient with suspected traumatic shoulder injury, comparing sagittal images of an UTE, T1 GRE, and FRACTURE MR-sequence (**A**–**C**) to a conventional CT scan (**D**). Note the detailed bone trabecula in the gradient echo sequences T1 GRE and FRACTURE (**B** and **C**) as well as the good bone to soft tissue contrast of the UTE sequence (**A**) compared to CT (**D**). Further notice the thickening of the cortical bone in the T1 GRE and FRACTURE sequence compared to the UTE sequence (white arrows)

Compared to the T1 GRE and FRACTURE sequences, distance measurements on the UTE sequence, in particular the glenoid bone width, anterior straight line, and glenoid bone loss, correlated best with conventional CT (Fig. 6). This might be due to the fact that the UTE sequence is less affected by susceptibility effects which expand the low signal regions next to the cortical bone and might lead to an overestimation of the cortical thickness. In contrast to the T1 GRE and FRACTURE sequences, the UTE sequence is based on a radial stack of stars readout starting in the center of the k-space which creates a fat blurring rather than a water/fat shift and might also contribute to the more accurate measurements.

As major limitation, UTE sequences require long scan times and are SNR limited. In order to reduce scan time and increase SNR, anisotropic voxels with comparable thick slice thickness were employed. There are different approaches for UTE imaging that allow to decrease the scan time, such as spiral trajectories and twisted or cones projection methods [7, 21, 32]. A 3D acquisition of high-resolution UTE sequences within reasonable acquisition times would significantly improve the use of this technique and should be investigated in further research. As further limitation, the intensive pulse sequences used in this study were optimized for newer gradient coils on a 3-T MR scanner, which might not be available everywhere and might be limited to certain centers. Application of these sequences on low performance gradient systems would most likely result in longer TRs and longer scan times. ZTE imaging was not available at our institution and therefore, the comparison between UTE and ZTE imaging was not possible although this comparison would be of interest, since these techniques are based on a similar technical concept. Patients with metal implants were not included into this study due to the sensitivity of the gradient echo sequences to metal artifacts. UTE imaging has potential advantages over the T1 gradient echo sequence in the presence of a metal implant, since UTE imaging is less prone to susceptibility artifacts. Further investigations are needed in order to evaluate these sequences with metal implants in order to assess the value, especially of the UTE, in a postoperative setting.

Finally, our results demonstrated that the evaluation of the shoulder joint in an acute trauma setting is feasible and accurate compared to conventional CT using CT-like images based on a T1 GRE, UTE, and FRACTURE sequence, potentially sparing the need for a conventional CT scan and the associated costs as well as radiation exposure.

### Supplementary Information

Below is the link to the electronic supplementary material.

Supplementary file1 (PDF 84 KB)

## Abbreviations

FOV: Field of view
FRACTURE: Fast field echo sequence resembling a CT using restricted echo-spacing
SNR: Signal to noise ratio
T1 GRE: T1-weighted spoiled gradient-echo sequence
TE: Echo time
TR: Repetition time
UTE: Ultra-short echo time sequence
ZTE: Zero echo time sequence

## Declarations

### Guarantor

The scientific guarantor of this publication is PD. Dr. med. Alexandra S. Gersing.

### Statistics and Biometry

No complex statistical methods were necessary for this paper.

### Study subjects or cohorts overlap

No overlap

### Methodology

- prospective
- case-control study
- performed at one institution
