# Supplementary material for: Assessment of glenoid bone loss and other osseous shoulder pathologies comparing MR-based CT-like images with conventional CT
Source: Eur Radiol. 2023 Jul 15;33(12):8617–26. doi: 10.1007/s00330-023-09939-9 (PMC10667374; doi:10.1007/s00330-023-09939-9)

Assessment of glenoid bone loss and other osseous shoulder pathologies comparing CT-like MR-images with conventional CT

ELECTRONIC SUPPLEMENTARY MATERIAL

Supplementary Figure 1

Complex scapula fracture of a 23-year-old patient after acute trauma showing sagittal images of a conventional CT scan (**A**) compared to inverted CT-like images acquired using UTE **(B)**, FRACTURE **(C)** and T1 GRE sequences **(D)**. Note the accurate presentation of the facture borders on the CT-like MR sequences compared to CT (white arrows).

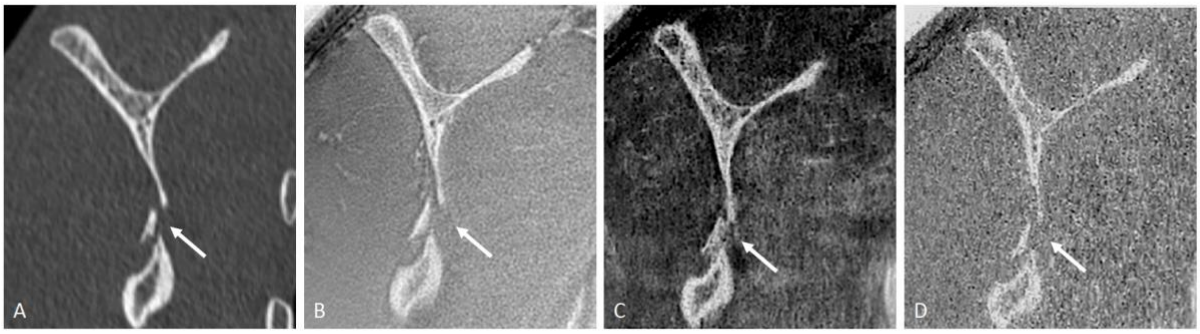

Supplement: Supplementary file 1 — Supplementary file1 (PDF 84 KB) [file 330_2023_9939_MOESM1_ESM.pdf]
